# Digital evidence exceptionalism? A review and discussion of conceptual hurdles in digital evidence transformation

**DOI:** 10.1016/j.fsisyn.2020.08.004

**Published:** 2020-08-28

**Authors:** Alex Biedermann, Kyriakos N. Kotsoglou

**Affiliations:** a University of Lausanne, School of Criminal Justice, 1015, Lausanne, Dorigny, Switzerland; b Northumbria University, School of Law, Newcastle Upon Tyne, NE1 8ST, United Kingdom

**Keywords:** Digital evidence, Weight of evidence, Probability, Evaluative reporting

## Abstract

Forensic science is currently undergoing a transformation and expansion to include modern types of evidence, such as evidence generated by digital investigations. This development is said to raise a series of challenges, both in operational and conceptual dimensions. This paper reviews and discusses a series of convoluted conceptual hurdles that are encountered in connection with the use of digital evidence as part of evidence and proof processes at trial, in contradistinction to investigative uses of such types of evidence. As a recent example raising such hurdles, we analyse and discuss assertions and proposals made in the article “Digital Evidence Certainty Descriptors (DECDs)” by Graeme Horsman (32 Forensic Science International: Digital Investigation (2020) 200896).

## 1 Introduction

In an editorial published a few years ago, Professor James Curran inquired about the intriguing question of why there is so much resistance against statistics in forensic science [25]. By statistics, Professor Curran did not primarily mean “the basic statistics requirement for any science degree” [25, p. 252]. He meant the principles and the logic of ‘‘evidence interpretation’’ [25, p. 252], that is the reasonable reasoning in the face of uncertainty. One would have hoped that mindsets averse to statistics improved over the last decade, not least because of specialist fora such as the triennal International Conference on Forensic Inference and Statistics (ICFIS, http://www.law.lu.se/#!ICFIS2020) and evaluative directives published by expert groups [80], professional associations [4], scientific committees [3] and collaborations between lawyers and statisticians from recognised institutions [e.g., 77]. To be fair, Professor Curran probably did not refer to forensic science as a whole, because this may have included himself. Rather, he referred to some quarters within forensic science that are reluctant to accept argumentative implications which derive from formal methods of reasoning, and which are applicable to forensic evidence evaluation (see also [10] for a further example). As we will exemplify and discuss throughout this paper, such quarters are also encountered in connection with the ongoing transformation and expansion of forensic science^1^ to include modern types of evidence, such as evidence generated by digital investigations. A specific source of concern is the use of digital evidence in evidence and proof processes at trial, in contradistinction to the use of such evidence in investigative proceedings, both of which involve a series of convoluted conceptual hurdles. The aim of this paper is to raise and discuss several of these hurdles, and to emphasise the timely relevant nature of this topic by reference to assertions and proposals made in recent scientific literature.

Specifically, we refer to a paper by Graeme Horsman [45] in which the author addresses a series of topics that we identify as conceptual hurdles in digital forensic science. In that paper [45] Horsman discusses means by which digital forensic experts may deal with uncertainty encountered in the course of evaluating and reporting results of digital evidence examinations. While we recognise this as a valuable aspiration, we disagree with several of the author’s assertions and conclusions. Further, as we will show throughout this paper, the author’s account of state of the art concepts and principles of forensic interpretation is, on many occasions, wide of the mark. The concern we share is that leaving the author’s account uncommented would mean to deprive the field from a balanced presentation of the properties of standard ways to cope with uncertainty, despite the fact that many of the points we shall make have been made before and that there is ample literature available on the topic. Thus, in a broader perspective, we also aim at inquiring about the reasons for the conceptual obstacles that we identify in digital forensic science, and in mindsets of commentators in this field. We will do so by looking beyond disciplinary borders, that is by uncovering parallels to controversies observable in scientific areas outside forensic science.

By way of an introductory example, Horsman writes that the efforts to quantify uncertainty, that is the hallmark of forensic science development since the second half of the last century, should be discontinued in the field of digital evidence. “It is argued”, he writes, “that attempts to quantify uncertainty should be abandoned” [45, p. 1].^2^ Further, he asserts that “at present there are no available satisfactory methods for achieving this [the quantification of uncertainty]”, and “suggests that attaining such methods may not actually be possible” [p. 1]. As we will show, these statements are at odds with what has been achieved in a variety of fields, including forensic science, concerned with the development and implementation of formal approaches to dealing with uncertainty. The purpose of the present paper is to critically examine and discuss these statements and others made by Horsman at various instances throughout his article. We will show that his approach towards formal methods of reasoning misconstrues the fact that human beings may have conceptual limitations (to enact formal reasoning methods) as a deficiency of the methods themselves. We will also show that Horsman’s proposal for a scale of “certainty descriptors” [p. 1] represents an instance of what, historically, is known as a collection of posterior-statements; to wit, direct statements (opinions) on propositions,^3^ based on particular evidence. For various reasons, both inferential and procedural in nature, this way of structuring propositions is unsuitable for forensic scientists^4^ operating at advanced stages of the legal process, in particular during evaluative reporting,^5^ and is incompatible with (domestic) legal provisions.

Within a broader perspective, Horsman’s diagnosis that the field of digital forensic science ‘‘diverges from other forensic-sub disciplines [sic]’’ [p. 2] and, in effect, digital evidence practitioners do not need to quantify their uncertainty, can be seen as a form of *intellectual exceptionalism*. This specific claim of exceptionalism and its potential drawbacks merit attention.^6^ Firstly, because Horsman’s paper as such does not convey a reliance on extensive research regarding key topics, such as probability. Secondly, the paper’s understanding of the structure of legal orders, especially the architecture of what we would call – at the cost of oversimplification – the adversarial criminal process, is problematic. The paper’s basic claim, i.e. the alleged need to eliminate subjectivity, is questionable in its assumptions and proposed methodology as a means for resolving practical problems in the administration of criminal justice. It merely adds to the uncertainty.

Our paper is structured as follows. Section 2 starts by providing a brief overview of the main critiques that Horsman formulates against quantitative approaches to measuring uncertainty. These critiques will be analysed and discussed in a series of subsections by exposing the feasibility, the nature and the purpose of the standard (scientific) measure of uncertainty, i.e. probability. In turn, Section 3 exposes the importance of distinguishing between investigative and evaluative modes of thinking and the implications of this distinction on the logical form of statements provided forensic examiners. Our argument here will be that different thinking-styles or, more formally, different methods of inference, require different forms of behaviour and actions, and different forms of expression of opinion. Section 4 examines Horsman’s non-quantitative proposal for capturing uncertainty, called Digital Evidence Certainty Descriptors (DECDs). We will show that the suggested DECDs framework replicates the design of conclusion-scales previously proposed in other forensic branches and, thus, is inevitably affected by the shortcomings of these concepts exposed in existing literature. We will show that the descriptive nature of DECDs make them unsuitable by design for interpretation at the evaluative stage because, by definition, interpretation is an analytical process that goes beyond mere description. Section 5 provides a summary of the principal discussion points and embeds these within a wider perspective. This section will argue that the successful extension of forensic science to include digital evidence hinges upon the field’s proper understanding of foundational aspects regarding the measurement of uncertainty (using probability) and the field’s commitment to the precepts of evaluative reporting in forensic science.

## 2 On the existence and feasibility of a (scientific) measure of uncertainty: the nature and assignment of probability

### 2.1 Horsman’s approach at a glance

The most visible thread in Horsman’s paper is his critique of formal methods of reasoning. As is illustrated by the following quotes, Horsman questions both the applicability of formal methods of reasoning in the context of digital evidence, as well as their existence:

• In his conclusions, he briefly mentions ‘‘mathematical methods for quantifying evidence’’^7^ [p. 8], but then asserts that there is ‘‘an absence of such methods’’ [p. 8]. This claim is demonstrably false, as we will show below.
• Elsewhere, Horsman seems to acknowledge the existence of formal methods of reasoning and their use in other branches of forensic science, but questions the applicability of these methods in digital forensic science. For example, on page 2, he writes that ‘‘many other traditional forensic science disciplines are encouraged to describe the weight of their evidence in some form of quantifiable measurement/expression’’, but that ‘‘at present, there are (…) no satisfactory ways to achieve this’’ in digital forensic science.^8^

The author offers no tangible warrant for his claim that formal methods of reasoning cannot be enacted in digital forensic science. He makes occasional reference to other known sceptics, such as Lund and Iyer [66]. However, this is ineffective as an argument because key claims of the latter have been refuted.^9^ Though the author rightly observes, factually, that formal methods tend not to be used in digital forensic science (‘‘this is rarely done in DF’’ [p. 2]), he also presents the stronger claim that his ‘‘work suggests that attaining such methods may not actually be possible due to the intricacies of digital data and the difficulties involved with the fine-grained interpretation of events’’ [p. 1]. This amounts to turning down normative requirements and the demand for a coherent framework which holds various practices together [15]. The argument here seems to be that the nature of digital forensic science is inherently different from that of other forensic fields, and hence excludes it from the application of formal methods of reasoning. The following assertion represents a further instance of what we call here exceptionalism: ‘‘It is proposed that achieving a scientific mechanism for quantifying digital evidence may not actually be feasible due to the nature of digital evidence and investigations of this type (…)’’ [45, p. 3].

Horsman presents these assertions as though it were clear what exactly it is in the ‘‘nature’’ [p. 3] of digital forensic evidence/science that makes this field unsuitable for the application of formal methods of reasoning, thus leaving his claim essentially unsupported.^10^ The author may object to this point by arguing that his article invokes the unavailability of (hard) data as a reason for the impossibility of using formal methods of reasoning (‘‘the lack of past case recording for statistical and learning purposes’’, ‘‘lack of any predefined statistical data’’ [p. 3–4]). This, however, would be an unfruitful defence because we would then need to presuppose that numerical input is a necessary requirement for formal methods of reasoning – which, clearly, is not the case [e.g., [11] (see also Section 2.3).

Horsman also raises the concern of subjectivity in relation with probability (‘‘The use of subjectivity in determining the weight of evidence will naturally create a level of uncertainty, but this is often a requirement for probabilistic methods.’’ [p. 3]), but abstains from explaining what exactly subjectivity means and does not mean in this context.^11^ Instead, Horsman aggregates both formal (i.e., probabilistic or quantitative) methods of reasoning and the colloquial use of the term subjectivity in a single category along with terms such as ‘misleading’ and ‘arbitrary’. This is illustrated by the following quotes:

• ‘‘However, any attempts to shoe-horn in such methodologies to DF where their application may not actually be feasible may be of greater detriment to this field, and provide an artificial ranking of evidence which may be misleading.’’ [p. 3]
• ‘‘(…) any weighting which is assigned by the practitioner which is derived purely from their own personal views is arbitrary, as previously stated, and is dangerous in terms of how a court may value such descriptions of evidence.’’ [p. 4]
• ‘‘A lack of any predefined statistical data regarding the likelihood of Action A or B being responsible when both remain possible means that quantify [sic] the likelihood of each is a challenge. The problem is that any assignment of likelihood to either Action to be responsible is arguably arbitrary and therefore meaningless and unreliable. Arguably, in this case it may be misleading to attempt to quantify the apparent uncertainty regarding which Action is responsible.’’ [p. 4]
• ‘‘(…) the inconsistent and subjective weighting of evidence.’’ [p. 8]

These are stark assertions, but as we will show below, they misrepresent the meaning of subjective or personal probability and its role as part of formal methods of reasoning. This is a hindrance for an informed discourse on the topic and amounts to an unsubstantiated reproach against formal (i.e., probabilistic) methods of reasoning. This calls for a clarification.

### 2.2 Uncertainty and probability

It is helpful to start our discussion with a clear statement of the notion of uncertainty. Although this may seem unnecessary, because the term is almost ubiquitously used in everyday language, insisting on two related but distinct components to the term uncertainty will be beneficial for the understanding of subsequent argument. First, in a nutshell, uncertainty means ‘‘the extent of our own knowledge and ignorance’’ [30, p. xvi]. Note that the words ‘‘our own’’ are central here. That is, what may be uncertain for one person may not be for another, because of differences in their respective bodies of knowledge and background information (this does not rule out that, sometimes, these may be coextensive for all practical purposes). Second, uncertainty relates to an aspect of the real world, although it is not – following the point made in the previous sentence – a feature of the world that exists independently of a human observer. In short, the focus is on ‘‘(…) your ([i.e.] the reader of these words) uncertainty about the world” [30, p. ix]. So, uncertainty is all about ‘‘*being* uncertain about something’’ [30, p. xv, *emphasis added*] of the present, past or future. By now, the attentive reader should have noticed a first key point: dealing with and, in particular, measuring uncertainty amounts to coping with a human condition; i.e., strength of belief in the truth or otherwise of a proposition of interest. Hence, it is little surprising that the process of dealing with uncertainty involves personalistic traits. One may wonder, thus, why there is so much discomfort among scientists against the personalistic nature of the measure of uncertainty. Denying this nature essentially amounts to denying reality. We discuss possible reasons for this reaction below.

The questions now are: How can one measure something, such as uncertainty, which is inherently personal? How can yours, ours, anybody’s uncertainty be measured? Note that the scientific approach to these questions places an emphasis on *measurement*, because ‘‘[…] it is by measuring things that we know them.’’ [61, p. 13]. The question of how to measure your^12^ uncertainty is widely covered in literature, which is why we will only mention a few core principles here, and give references in which more detailed presentations can be found. First and foremost, probability is not a question of relative frequency ideas^13^ because there is nothing repeatable here. There is only a single, non-replicable event.^14^ Second, measuring uncertainty is an entirely general question that does not depend on the area of application, be it (digital) forensic or one pertaining to an aspect of daily life, i.e. formal or informal. Third, measuring uncertainty is not fundamentally different from measuring in other areas of science: measurements are made by reference to a standard.^15^ The notion of ‘standard’, though, warrants a comment. As has been pointed out in philosophical literature, extreme vagueness is mainly a problem of measurement [6, p. 190–192], leading each individual to deploy his or her own understanding by reference to his or her own inner sensations. To measure, one needs a reference (point) to serve as a standard (e.g., of proof) or, alternatively, a so-called canonical instance (or, sample). Coherence and rationality demand that a fail-safe standard of measurement is to be applied in every instant case. It is deeply undesirable and deleterious especially for the coherence of a legal system to tolerate a practice wherein different expert witnesses assess and articulate uncertainty in radically different ways. For, ultimately, we would not apply methodological rules in order to structure and measure personal beliefs, and quantify uncertainty; on the contrary, we would end up using the respective inner sensation in order to fix the supposed measurement of uncertainty. Whereas we should let the standards of rationality determine the measuring instrument, the opposite is the case in Horsman’s article. His approach leads inexorably to the situation where the process of capturing uncertainty is treated like a flexible mass, rather than by reference to a canonical instance. What is more, Horsman’s approach dispenses with measurement altogether by resorting to mere description, which is insufficient for dealing with uncertainty.^16^

Turning now to the measurement of uncertainty using probability, we shall again keep a concise account. One among several ways to conceptualise probability is to say that your probability for an event that is uncertain for you is m/n if, for you, the uncertainty is *judged* to be the same as when drawing a ball at random from a bag that contains *n* balls, of which *m* are black, and that the single ball withdrawn turns out to be black [e.g., 62].^17^ There must be an urn with a proportion of black balls (different from zero and one) so that the probability of drawing a black ball is such that the uncertainty is, for you, judged to be the same as that which, for you, endows the event of interest. Probability, thus, our measure of uncertainty, is a number between zero and one.

In relation to this, a few comments are in order. First and foremost, the above conceptualisation is about the feasibility in principle of measuring uncertainty. It must not be confused with the applicability of the measuring device. Applicability, which may indeed not be easy, is a distinct matter in its own right. It may not be easy for you to decide which proportion of black balls reflects your uncertainty regarding the event you are contemplating. Yet, this is of no detriment to the fact that, fundamentally, the measuring device is simple and intuitively understandable. Second, to be clear, the suggestion here is not that we ought to keep, or even bring to court, a real (i.e., physical) bag or urn filled with balls to be used for measuring our uncertainty about events of interest. The device is a conceptual one, used merely to clarify precisely what we mean by ‘‘being uncertain’’ [30, p. xv]. There are other such devices, studied widely by psychologists.^18^ Third, only your uncertainty is of importance: whether the actual truth-state of the uncertain events is known or knowable by others, for example, has no bearing on your uncertainty [30].

### 2.3 Relation to statistics

Attentive readers may have observed that, so far, we have not yet mentioned the field of statistics. They may even wonder whether the above conceptualisation is ignoring statistics (and data analysis) by defining statements of personal belief as the overriding mode of capturing uncertainty. The contrary is the case. The device introduced above is merely providing the starting point for explaining principal aspects, such as probabilities being given by numbers in the range between zero and one. While, in its widest sense, ‘‘statistics is essentially the study of uncertainty’’ [63, p. 294], some tend to think of statistics as the field that applies whenever there is data to be used for inference. That is, the coherent revision of an initial probability (regarding an uncertain event of interest) in the light of new data. This represents an entire field in its own right [e.g., 2, 73] which is beyond the scope of the discussion here. The current discourse is at a much more basic stage: the nature and formal expression of uncertainty *irrespective* of whatever data there may be to help appreciate it. More so, in some situations – often times in forensic science – there may be no suitable data in the instant case. Yet, at the end of the day, we remain with uncertainty about the truth of a contested event. It is of interest then to conceptualise uncertainty about this event in a defensible manner.^19^ If we cannot do this, or we are unwilling to admit that uncertainty measurement devices exist and can, in principle, be enacted, any more sophisticated study of uncertainty (e.g. more advanced statistical implementations and the use of specific data), cannot even get off the ground. However, this is what Horsman’s paper is advocating: it contests the existence and applicability of quantitative measures of uncertainty (e.g.: ‘‘an absence of such methods’’ [p. 8]). By doing so, it deters the field (of digital forensic science) from apprehending these measures in a beneficial way.

We saw above that the measurement of uncertainty using probability can be stated in simple terms. It can be conceptualised in a clear and concise way that should be understandable for general audiences, including ‘‘business executives, (…) politicians, as well as scientists’’ [61, p. vii]. In short, for ‘‘(…) anyone who is interested (…) and (…) prepared to take the trouble to follow a reasoned argument’’ [61, p. vii]. But how, then, can it be that members of the forensic science community, let alone peer reviewed journals in this area, adopt probability-averse positions? This is an intriguing question. While it may be one for sociological research to investigate, it is stimulating to consider how statisticians have reflected on this issue:

‘‘The truth of the matter is that people dislike simple problems: they like to take refuge in complicated ones where the inadequacies of their procedures are difficult to challenge because of the obscurity generated by complication. As has been said, ‘Practical decision-makers instinctively want to avoid the rather awful clarity that surrounds a really simple decision’. The reply to the accusation of guessing at probabilities (…) is simply that if you can’t do simple problems, how can you do complicated ones? To which a reply is: there are complicated problems that people can solve without being able to solve the simple ones that underlie them – for example, riding a bicycle. Is decision-making like riding a bicycle? I think not. People ride bicycles by repeated practice until, one day, it comes to them; they can do it, they never forget, and they don’t know how they do it. None of these notions easily carry over to decision-making.’’^20^ [61, p. 65]

A further way in which probability scepticism in (digital) forensic science may be understood is to see it as a result of abstaining from looking beyond disciplinary borders. This prevents one from understanding that the nature of, and the key to the conceptual problems faced by forensic scientists do not lie in forensic science itself, or in (digital) forensic science exceptionalism. Instead, the ability to cope with these problems hinges on our capacity to absorb the results of developments in the areas of philosophy of science, mathematics, statistics and the broader interdisciplinary research fields of decision science. As an example, it is interesting to note that even among statisticians it is considered a fundamental challenge to properly conceive of a starting point in their field [53]. Thus, for forensic scientists trying to incorporate the same fundamental insights may be even more demanding. The seriousness of this challenge should be clear. We doubt that it makes sense for (digital forensic) scientists to attempt to conceive their own ‘theory’ for reasoning under uncertainty. Similarly, forensic biologists would not be well advised to do without the advances of modern genetics, for this would imply the rejection of current DNA profiling technology. History of science unmistakably issues the warning that scientists who follow the King of Hearts’ advice and ‘‘begin at the beginning’’ by setting their own first principles inexorably run into difficulties. They will not benefit from the level of sophistication in their field and be hard pressed to do any meaningful work [44]. Yet this currently appears to happen in some parts of digital forensic science.

### 2.4 Misconceived subjective probabilism and spurious objectivism

Horsman’s ‘‘work examines the issues surrounding the quantification of DF examination results’’ [p. 2]. His key assertion is that pressing too much on implementing a quantitative approach, i.e. more than he thinks is actually feasible, is counter-productive: this ‘‘(…) may be of greater detriment to this field, and provide an artificial ranking of evidence which may be misleading’’ [p. 3]. In the same vein, Horsman describes the expression of (subjective) probabilities (‘‘assigned probabilities based on experience and subjective judgement’’ [p. 1]), that is the ‘‘inconsistent and subjective weighting of evidence’’ [p. 8], as subject to criticism, indeed inferior to ‘‘(…) objective measures [which] are considered more favourable’’ [p. 1]. He asserts that the term subjective (in the sense of ‘‘personal views’’ [p. 4]) means or implies being artificial, misleading, arbitrary, meaningless, unreliable or even dangerous. Note that Horsman raises all of the latter words at various instances throughout his paper, either individually or in combination; see either the quotes given above as well as the following two excerpts: ‘‘any assignment of likelihood (…) is arguably arbitrary and therefore meaningless and unreliable’’ [p. 4] and ‘‘any weighting which is assigned by the practitioner which is derived purely from their own personal views is arbitrary (…) and is dangerous in terms of how a court may value such descriptions of evidence’’ [p. 4].

These are severe words against a single notion. But, as it stands, this account of subjective (personalistic) assessments (i.e., probabilities) does not properly represent what personal probability is about. Again, the point has been made repeatedly in literature and current recommendations, but the fact that it continues to be misstated warrants a brief recapitulation of a few key points. Horsman’s perspective is predicated on the assumption that subjectivism is tantamount to deliberate guesswork, elsewhere called ‘‘*unconstrained* subjectivism’’ [17, p. 478, *emphasis as in original*]. This, however, is not how subjective probabilists who are serious about their scholarship understand and advocate the notion of subjective (i.e., personal) assessments (of probability). First and foremost, they do not equate subjectivism with arbitrariness: the liberal^21^ nature of probability does not mean that one should feel free to assert any number, without justification. What is of interest is not *any* probability assignment, but a *justified* probability assignment.^22^ Clearly, the idea here is that ‘‘[w]e strive to make (…) judgments as dispassionate, reflective and wise as possible (…)’’ [27, p. 144]. We are asking for ‘‘[r]reasonable personal probabilities’’ [41, p. 1504], admitting to the reality that ‘‘[i]f we cannot require everybody sharing the same likelihoods, we can require everybody having *justified* likelihoods’’ [41, p. 1506, *emphasis as in original*]. Of course, this does not prevent some forensic scientists from providing questionable forensic testimony, e.g. in terms of categorical conclusions, or even sheer denial of the potential of error, but this does not invalidate the concept of personal probability – it ignores it. The ENFSI Guideline, for example, clearly states that probabilities ought to be viewed as ‘‘conditioned on the information available to the individual who makes a probability assignment’’ [80, p. 23]. Moreover, ‘‘[t]he basis for these assignments shall be documented on the case file’’ [80, p. 15], thus conforming to the requirement of justification as stipulated above. Specifically, the ENFSI Guideline states:

‘‘*personal* probability assignment is not arbitrary or speculative, but is based on a body of knowledge that should be available for auditing and disclosure. The forensic practitioner should not mislead the recipient of expert information as to the basis of the *personal* assignment, and the extent to which the assignment is supported by scientific research.’’ [80, p. 16, *emphasis added*]

Specifically, it is required that any assignment, wherever possible, *is* based on data, that is ‘‘the technical and empirical knowledge associated with a given trace type’’ [80, p. 19]:

‘‘data can take, for example, the structured form of scientific publications, databases or internal reports or, in addition to or in the absence of the above, be part of the expert knowledge built upon experiments conducted under controlled conditions (including case-specific experiments), training and experience.’’ [80, p. 19]

Note that there is a hierarchy stipulated here. Scientists cannot use a vague reference to training and experience (mentioned last) as a substitute to hard-wired scientific publications (mentioned first). To some extent, it is surprising that all of this needs to be stated so forcefully. In an ideal world, one would expect forensic scientists to care about the reasonableness and robustness of their statements, not least because their assertions expose them directly to scrutiny by their peers.^23^ Conversely, it also seems to go without saying that there is no suggestion, especially not in the ENFSI Guideline, that scientists ought to make assessments *at any price*, i.e. when they feel that a defensible assessment cannot be given. Thus, Horsman’s discourse about there being ‘‘attempts to shoe-horn in such methodologies in DF investigation’’ [p. 3] is alien to the principles and intentions of forensic evaluation and evaluative reporting.

We should also insist that criminal adjudication in England and Wales – i.e. the main subsystem – defines the activities of auxiliary forces (such as forensic science) by specifying aspects such as the expert’s duty to the court (see The Criminal Procedure Rules 2015, hereafter: CrimPR, Rule 19), the way that experts will deliver their scientific input (i.e. not in their accustomed laboratory environment, but in a court room where people wear wigs and robes, CrimPR, Rule 19.3) and, more importantly, the structure and content of the expert’s report (CrimPR, Rule 19.4). Experts will do what the court and methodological principles salient in scientific enquiry and adapted to practical purposes ask them to do: address the probability of the findings given the propositions and relevant background information, and remain silent on the probability of the propositions given the findings and background information. Opining, let alone deciding on questions of justice is not required or indeed permitted for forensic experts. Hence, Horseman’s language involving terms such as ‘‘factually prove a hypothesis’’, ‘‘conclusive fact’’, ‘‘100% proof’’ [p. 7] is unsuitable in principle for forensic scientists.

By dismissing the idea of quantification as a whole, and by advocating an exclusively qualitative (i.e., verbalistic) framework^24^ as an alternative, Horsman takes an extreme position. His reaction suggests that there is only either a fully quantitative mode of operation, or an entirely non-quantitative one. However, this is not the case. Probability is a flexible framework that can be applied at various levels of detail, invoking notions such as qualitative probabilistic reasoning [e.g., 79], orders of magnitude and sensitivity analyses [e.g., 11].^25^ And yet, a more radical reply to the aversion to quantification can be given. That is, rather than defending numbers – which is the position taken here – one could invoke the position according to which, numbers are not the primary objective, but coherence [69, p. 168]. Note, however, that this is different from Horsman’s position that seeks to keep out of digital forensic evidence, not only arithmetics, but the framework (of probability) as a whole.

Critics of subjectivism commonly invoke objectivism as the obvious solution, so does Horsman (‘‘concise representations (…) that a practitioner can objectively describe’’ [p. 6], ‘‘objective measures are considered more favourable’’ [p. 1]). This is predicated on the assumption that it is clear what objectivism means, let alone it were feasible. The dualism that lingers here is that if subjectivism is intrinsically inadequate, then its opposite, objectivism, must necessarily be good. However, the contrary is the case: no choice of a framework, method or procedure can be made without resort to at least some personal judgment [9,13]. Though widely ignored, this point has as abundantly been made, for example, by forensic scientists [34], statisticians [8,64], legal theorists [55] and philosophers of science [46].

A scientist’s assessment thus is inevitably personalistic. It relies on a body of knowledge and data which is specific to that scientist. By highlighting the necessarily subjective nature of their assessments, and by redirecting their efforts to clarifying the extent to which these assessments are, using the words of the ENFSI Guideline, ‘‘supported by scientific research’’ [80, p. 16], scientists can demonstrate that they take responsibility for their assessments and take the burden of justification seriously. Being prepared to expose the credentials of their assertions, to the best of their knowledge, scientists could become counter-examples for Lad’s observation that ‘‘[t]he modern conception of scientific method as an objective and value–free learning procedure is the source of the distrust and disrespect shown to science by many people today’’ [57, p. 441].

### 2.5 The inevitability of probability and probability as a decision

By sidestepping probability and proposing his own ‘‘language framework which defines the terms which should be used to describe a practitioner’s level of certainty in the evidence/digital content’’ [p. 6],^26^ Horsman makes a problematic suggestion. The proper way to capture uncertainty is, as we understand Horsman’s proposal, an activity that is unconstrained by formal requirements. That is, probability is just as good as any other notion, however intuitive, and that one may endorse it or not, comparable to a matter of personal taste. However, this view misconceives the nature of probability as a measure of uncertainty. Probability is all about ensuring (logical) coherence, hence it is neither arbitrary, nor could it be easily ignored – on pain of falling into incoherence. This is why it has been argued that ‘‘[o]ne cannot sit down and think up apparently reasonable rules (…) because one is not free to engage in the intellectual exercise of law creation. The laws are forced upon you. It is a case of the inevitability of probability. The laws ensure that several statements of uncertainty cohere.’’ [61, p. 37].

To be clear, the suggestion at this juncture is not that probability theory ought to be taught in the courtroom. The discussion here is about the way in which forensic scientists should make up their mind when dealing with uncertainty and assigning a value to their findings – prior to proceeding with evaluative reporting.^27^ The modest expectation is that this ought to be done in a reasonable way. Reasonableness, in this context, means conformity with reasonable rules of reasoning, i.e. probability.

Yet another way to understand the inevitability of probability is to recognise that, fundamentally, assigning a probability amounts to making a decision, for there is no probability unless one is being assigned. The question then becomes how to *decide* on a probability assignment in a way that is logically defensible, i.e. rational. What this means, in simple terms, is to understand and formally reconstruct the problem of reporting on imperfect personal knowledge in terms of probabilities, as a personal decision problem. There is a whole body of specialised literature on this topic that arose from the study of the question of how to assess, so-to-speak, the ‘goodness’ of probability assessors [e.g., 26]. These works, whose details go beyond the scope of this paper, build upon decision theory and operational devices such as scoring rules for supporting the assignment of personal probabilities [e.g., 18, 60]. Though rarely acknowledged in mainstream discussions about probability [65],^28^ it is relevant to emphasise here that the decisional perspective to probability has been referred to as the single most meaningful concept [29]. The relevance of mentioning this account for the purposes of the discussion presented in this paper is twofold. First, on a theoretical account, it provides a (further) method for measuring uncertainty,^29^ thus adding to the scope of scientific ways of measuring uncertainty – recall that Horsman denies the existence of such methods (‘‘given an absence of such methods’’ [p. 8]). Second, on an operational account, the understanding that probability assignment amounts to, ultimately, a decision is valuable because it reinforces the fact that the ‘problem’ of probability assessment does not rely in the method, but in the limitations of those who use it. Specifically, understanding probability as a decision most incisively reveals that probability assignment requires one to take personal responsibility for one’s assertions. However hard this simple reality is to accept, or to commit to, it is precisely what recipients of expert information consider a desirable property of evaluative reporting.

### 2.6 Likelihood and probability

When being uncertain about an event of the present, past or future, we commonly express this uncertainty in terms of probability. As mentioned in Section 2.2, probability is the measure of an individual’s degree of uncertainty; it is a state of mind, that is the degree of belief that a person holds concerning selected propositions of interest [29]. Horsman consistently avoids expressing himself in terms of the probability of an event of interest, especially the probability of observing evidence *given* a particular proposition.^30^ The latter is the key notion underlying the probabilistic measure of the value of evidence, known as the likelihood ratio [2]. Instead, Horsman uses expressions of the following kind, equating uncertainty with likelihood: ‘‘unquantifiable uncertainty/likelihood’’ [p. 3]; ‘‘one where Action B is not likely to be responsible for Result A and one where Action B is likely to be responsible for Result A’’ [p. 3]; ‘‘quantification of the likelihood of Action B’’ [p. 3]; ‘‘likelihood of Action A or B being responsible’’ [p. 4]; ‘‘Whilst potentially unlikely, it may not be possible to refute this action’’ [p. 4].

This raises the question of how meaningful it is to avoid the term probability (probable) by using likelihood (likely) instead. In statistical theory, a distinction is made between the terms likelihood and probability [e.g. 65], but unfortunately this distinction is often overseen or ignored in forensic science literature. At the same time, explaining the difference between likelihood and probability may be confusing when experience shows that in practice, especially legal practice, the two terms are largely taken to be synonyms. Notwithstanding, we shall make the difference here because it allows us to reveal a contradiction in Horsman’s framework and assertions.

For what it is worth, the distinction between likelihood and probability, in statistical terms, involves different targets. Technically speaking, likelihoods pertain to hypotheses, not evidence. One speaks of likelihood of a proposition for a given (fixed) item of evidence (or, in statistics, data). At the same time, the *probability* of the evidence given a proposition is called a likelihood (but: a likelihood of the proposition).^31^ This can be understood by considering the notion of *likelihood ratio*, that is the ratio of two *probabilities* of a given (fixed) item of evidence conditional on, respectively, each of two competing propositions. In short, thus, likelihood describes the probability of the evidence conditional on *different* propositions. What this distinction shows us in the context of Horsman’s paper is that when he expresses himself in terms of, for example, the ‘‘likelihood of Action A or B’’ [p. 4] (where A and B are propositions), then, in a technically strict reading, he refers to probabilities of some evidence conditional on, respectively, propositions A and B. However, the probability of the evidence given a proposition is an inferential target that he precisely seeks to avoid. This renders his critique of probabilistic evidence evaluation internally contradictory.

Thus, ultimately, it remains unclear which uncertainty, and measure thereof, Horsman exactly refers to: that of the findings (evidence), the competing propositions (i.e., explanatory accounts), or both. While definitional intricacies of technical language may be a reason for this vagueness, yet a deeper problem is a lack of clarity regarding the purpose of his analyses and discussion. The next section addresses this issue.

## 3 Clarity on the purpose: investigation versus evaluation

Besides conceptual shortcomings in assertions about methods for the measurement of uncertainty exposed in the previous section, Horsman’s paper involves confusion with regard to the distinct role in which forensic scientists may operate. This distinction has to do with investigation as compared to evaluation. Horsman writes that his paper deals with ‘‘the *interpretation and presentation* of results; the latter will be the focus here’’ [p. 1, *emphasis as in original*].

This raises the question of how this topic can be meaningfully tackled without acknowledging the foundational and vast works on case assessment and interpretation (CAI) [24, 49]. Broadly speaking, CAI sees investigation as being centred around questions such as ‘what happened?’ or ‘what (material) is this?‘. It may involve scientists suggest explanations for observations, or even suggest a ranking of competing explanations according to their relative plausibility [48]. A suspect (or, more generally, a person of interest) does not necessarily need to be available at this juncture. In contrast to this, the evaluative stage is fundamentally different. According to the ENFSI Guideline, *evaluation* of forensic findings becomes necessary when there are two competing versions of a contested event, typically brought up by adversarial parties at trial, and when it is of interest to assess the value of the forensic findings in helping to discriminate between these two competing accounts [80]. Moreover, a person of interest is typically available at this point and materials seized in relation with that person is the object of forensic examinations. Most importantly, at the evaluative stage, the proper role of forensic scientists is no longer to opine directly on propositions; instead, they must concentrate on assessing the value of the findings, that is focusing on the probability of the findings *given* a pair of competing propositions. Often, these propositions map, though not necessarily so, on the propositions brought forward by the parties in litigation. These propositions must be taken as a given. Examiners should not lose sight of the fact that their role is limited to providing assistance to others, i.e. the triers of fact at a criminal court.

Horsman’s paper is, however, concerned with the investigative-type issues rather than evaluative-type issues. He describes the role of examiners as follows: ‘‘(…) the practitioner must determine if it is possible to identify which actions may be responsible for the evidential result’’ [p. 3]. Clearly, in the CAI framework, this can be recognised as an instance of hypotheses generation. Horsman also proposes a scheme to provide an appreciation of the extent to which the various evidential settings involve uncertainty. In the context of the CAI framework, this would amount to ranking the various explanatory accounts in terms of their posterior probabilities.^32^ But at this point, Horsman’s framework stops, suggesting that an investigative opinion is all that can be given, and that it is ready to be carried over to more advanced stages of evidence and proof processes. But, this is not the case. Recall that in the context of investigation, examiners may be allowed to opine on explanatory accounts, so Horman’s generation and ranking of hypotheses may be fine for exclusively investigative purposes.^33^ Notwithstanding, it is unfit by design for evaluative purposes at trial because, as explained above, the requirements at this latter stage of the legal process are fundamentally different from those of investigation.

At this juncture, let us recall that the important difference lies in the form of the inference that will address, in a logical, justifiable way, the specific questions in a case. The starting point for CAI is: what are the questions/issues? Then the expert has to group these questions/issues into two categories and decide whether she has the necessary knowledge, competence and licence to provide direct answers to the questions and/or the necessary knowledge, competence and licence to provide an evaluation of the weight of evidence in favour of one of two (or more) competing propositions. The two broad forms of question/issue and inference (investigative and evaluative) may well overlap and within any one case. The expert has to be acutely aware of the difference and the interplay.

Having clarified the different aims and, related to that, the content, form and logical structure of examiners’ conclusions and reports (i.e., opining on explanatory accounts in investigation vs. focus on evidence given propositions in evaluation), it is now possible to review Horsman’s account from a broader perspective, to examine whether his aversion to the use of probability is well grounded. In essence, what Horsman is arguing for is the sorting out of viable accounts as to how digital forensic findings (evidence) came into place. As noted above, established interpretative accounts in forensic science, i.e. CAI, have never contested such an activity for forensic examiners. Quite to the contrary, CAI has explicitly reserved room for this, in the form of *investigative opinions*. Most importantly, and this is the critical point, CAI has never insisted on the fact that such investigative opinions must strictly and systematically be structured in strictly probabilistic terms.^34^ Thus, when Horsman criticises probabilism in the context of investigation, he essentially argues against a straw man. The probabilism that Horsman addresses, in particular the concept of probability of the evidence given the proposition, is – in terms of CAI – part of evaluative procedures, not necessarily investigative settings.

This confusion is unfortunate, essentially because an acknowledgment of CAI literature should have clarified matters right away. As an aside, this discussion reveals a deeper problem of current forensic science research: the problem of disregarding fundamentals. The difficulty here lies in balancing the use of concepts from outside forensic science. Where this difficulty is avoided, resorting to (digital) forensic science exceptionalism should come as no surprise. The importance of attaching to fundamentals, especially in forensic interpretation, has long been recognised [31]. Thus, studying and acknowledging foundational literature, in particular its key messages [e.g., 33],^35^ should help us avoid misconceptions that can grow out of ad-hoc theorising. While a lack of appreciation of foundational literature can also be a problem in other areas of science [36], one would hope that forensic science should aim at doing better, not least because its pretension is to serve the judiciary and because scientists’ conclusions may adversely affect innocent defendants or, alternatively deprive the victims of crime the justice that they deserve.

For the sake of argument, let us suppose now that the analysis in the present paper is wrong and that Horsman’s criticism indeed targeted evaluative (rather than only investigative) reporting and the impossibility to address questions regarding the probability of the evidence given the proposition. More specifically, suppose that even the most sophisticated digital forensic science expert could not address, for whatever reason, the question of the probability of the evidence given the propositions of interest in an instant case. That would be a very useful finding. It would mean that the value of the evidence could not be assessed,^36^ and hence the evidence should be kept out of evaluative proceedings (at trial) altogether. The reason for this is that such an item of evidence is simply uninformative,^37^ and trying to adduce it would be a waste of time and resources. Such an item has no (probative) value. It would provide no assistance to anyone asked to decide which of the competing propositions debated at trial is proven in view of the requisite legal standard of proof – regardless of any use made in investigation. This shows that solving an investigative problem is not tantamount to solving an evaluative problem.

## 4 Digital Evidence Certainty Descriptors (DECDs): the numbers trap and the intricacies of qualitative abstractions

### 4.1 Fundamental design problems of DECDs

Horsman’s proposed solution to the alleged inexistence and unfeasibility in principle of methods for quantifying uncertainty is a range of verbal descriptors, seeking to convey the extent of (un)certainty (‘‘the use of language as a way of expressing ‘certainty’ (…) and clarity in regards to what the language means when used’’ [p. 6]), called Digital Evidence Certainty Descriptors (DECDs):

‘‘(…) this work proposes that DF opts for the use of descriptors to indicate apparent uncertainty but refrain and accept we cannot yet quantify it. Rather than strive to numerically quantify evidence weightings, it is proposed that a more viable option is to harmonise the language practitioners use to express certainty in their findings.’’ [p. 6]

The digital evidence certainty descriptors involve six qualifiers, ranging from ‘‘conclusive fact’’ (‘‘proof in relation to a given scenario’’ [p. 7]), various intermediate levels such as ‘‘conceivable’’ (‘‘the lowest form of certainty’’ [p. 7]), to ‘‘impossible’’ (‘‘events which cannot possibly occur’’ [p. 7]). It is worth mentioning that Horsman explains his scale by resorting to numerical expressions. For example, the descriptor ‘‘conclusive fact’’, should apply when ‘‘we are looking at 100% proof’’ [p. 7]. But since DECDs involve ordinary words, trying to *explain* let alone define them raises a problem on legal grounds. Courts in England and Wales are at pains to stress that it is a general principle of English law that ordinary words are notions on which the fact-finders will decide based upon their own experience of ordinary life. Experts should therefore refrain from opining on their meaning, for they are almost by definition non-experts on the meaning of ordinary words. Lord Hughes in *Golds* makes a similar point when he highlighted the principle of ‘‘leaving an ordinary word alone’’.^38^

The choice of the verbal terms in the DECDs scheme, their number and definition shall not be discussed here. Recalling discussion presented in Section 2.4, it shall suffice to notice that this range of descriptors represents an example for a personal (rather than an objective) choice of framework. In what follows, the logical structure of the proposed evidence certainty descriptors will be examined, revealing a fundamental design problem.

As their name suggests, the proposed verbal descriptors (DECDs) seek to qualify the (extent of) certainty, but the target of this description is confusing. DECDs are supposed to concentrate on the evidence (i.e., ‘‘conveying when uncertainty exists in a set of digital findings’’ [p. 1]), yet the definition given in Table 1 (in Ref. [45]) on page 7 is consistently framed as conclusions regarding hypotheses (i.e., competing explanatory accounts): ‘‘proof in relation to a given scenario” [p. 7], ‘‘the proposed hypothesis is not disproved’’ [p. 7], ‘‘conveying how reliable they deem any resulting hypotheses from (…) digital investigations’’ [p. 8]. Hence, it is not clear whether Horsman is qualifying uncertainty in relation to the evidence *given* various competing propositions, or the uncertainty affecting propositions *given* (i.e., in the light of) particular evidence. Following arguments exposed in Section 3, only the former is suitable for evaluation while the latter may be acceptable for investigative purposes.

If the intended design of DECDs is to qualify the evidence and, at the same time, suggest a conclusion about competing propositions (or the discriminative capacity regarding competing propositions), then DECDs warrant a further comment: the lack of an argumentative basis for the claim of going directly from observations to conclusions. The history of forensic science has seen numerous attempts (ineffective on logical grounds) of designing conclusion formats of the kind ‘if *α* is observed, conclude *β*’. For example, the AFTE (Association of Firearm and Toolmark Examiners) theory of identification stipulates that when there is ‘‘sufficient agreement’’ [23, p. 287] (i.e., a given type of observation is made), then this ‘‘enables opinions of common origin to be made’’ [id.] (i.e., a statement regarding the truth or otherwise of a particular proposition). For a critical discussion of attempts to design a similar conclusion scale in the area of forensic shoemark examinations, see Ref. [21]. The fundamental problem with these suggestions is that they oversee that support for a particular proposition, as provided by evidence, is an inferential concept that requires one to observe, on pain of falling into incoherence, certain logical principles: in essence, these come down to assessments that take the form ‘probability of the evidence given the proposition’ [1]. There is no known proper method of inference that justifies a procedure whereby predefined qualifiers, to serve as conclusions to be reported, are to be picked from a list consisting of various verbal descriptions (i.e., definitions of qualifiers).

One may object to this critique by arguing that there is no intention to endow DECDs with any formal inferential mechanism, only with a purely descriptive purpose. After all, their name contains the word ‘descriptor’, and throughout his paper, Horsman widely uses the words ‘describing’ and ‘description’ (e.g., ‘‘DECD is designed to ensure that the language practitioners use to *describe* when uncertainty is present in an examination is consistent’’ [p. 6, *emphasis added*]). However, if that is the case, this falls short of the stated purpose. Recall that Horsman’s paper stated its purpose as follows: ‘‘*interpretation and presentation of results*; the latter will be the focus here’’ [p. 1, *emphasis as in original*]. The point we intend to make here is that interpretation and description are different topics. Conventionally, description has to do with capturing, by an observer, perceivable aspects and properties of a descriptum, that is the entity to which a description refers. Interpretation, in turn, is different in the sense that it is *not* descriptive, but analytical, and involves inference. As per definition, the target of interpretation, that is the meaning of evidence, is *not* an inherent property that could be captured in a descriptive sense. Arriving at an interpretation through description, as is the pretence of DECDs, thus amounts to a contradiction in terms. If one wishes to *describe* the strength (or meaning) of evidence, one first needs to work it out, which can only be achieved by interpretation, *not* description. Pretending the contrary would amount to what Salmon has called ‘‘epistemological magic’’ [71, p. 66].

To be clear, interpretation seeks to work out the meaning of an interpretans through inference, that is the use of a reasonable (i.e., coherent) method of reasoning. The logical structure of such methods of reasoning relies on the formal method for dealing with uncertainty whose existence and/or applicability Horsman questions (see quotes given in Section 2.1). In conclusion, thus, Horsman’s framework is neither analytical in an inferential sense, nor interpretative, in the proper senses of these terms. It cannot be because it abstains from the scientific method of dealing with uncertainty, which is probability.

### 4.2 The eternal ‘could have/be’

The framework of DECDs contains several instances of the use of language involving the expression ‘could have/be’:

• ‘‘Multiple actions *could be* responsible for same output.’’ [p. 3, *emphasis added*]
• ‘‘Possible that this information *could have been* the result of a number of actions such as part of a legitimate website which has been cached and deleted without a user ever visiting it or it could *have been* sent to the device via spam emails which have since been deleted.’’ [p. 7, *emphasis added*]
• ‘‘It may be ‘*Conceivable*’ that a suspect *could have* browsed the apparent URL’’ [p. 7, *first emphasis as in original*, *second emphasis added*]

The expression ‘could have/be’ is particularly unsuitable for use by forensic scientists for a number of reasons and has, thus, repeatedly been advised against. In what follows, we shall largely rely on other authors in order to insist on the fact that none of our arguments is fundamentally new.

First and foremost, the expression ‘could have/be’ induces scientists to opine on the plausibility of propositions (i.e., the events that ‘could have’ led to the findings) which, as exposed in Section 3, is not appropriate for evaluative reporting.^39^ It has also been noted that ‘‘[t]he phrases ‘‘consistent with’’ and ‘‘could have’’ appear still widely used in the forensic science community because they lead to a fairly easy life: no real interpretation is required, and often the statements made are little more than statements of the obvious. However, science is about *understanding* (…)’’ [35, p. 10, *emphasis as in original*].^40^ Further, it is difficult to keep track of how different recipients of expert information align in their understanding of expressions such as ‘could have’ (see e.g. [47, p. 82] for an example involving the range between 1 in 3 and 1 in 3 million).

In combination, the above observations reveal a key insight: avoiding quantitative expressions through the use of qualitative verbal expressions will leave recipients of expert information clueless about the strength of one’s message, to the point of being a statement of the obvious.^41^ As such, the use of the expression ‘could have/be’ in forensic science provides a textbook example of what in literature in the philosophy of probability has long been recognised as hollow expertise:

‘‘(…) the answers provided by the experts, when formulated in words, tend to be rather elusive. Typically experts give evaluations like: ‘‘It is almost certain, but not sure; indeed it might not be the case.’’ and so on. They say and unsay in the one breath, not to risk too much. The result is that it is never quite clear what their answers mean. The expert tries to speak in such a way as to secure himself that, whatever happens, one cannot tell him that he was wrong. On the contrary, if questions are asked in such a way as to obtain a probability value as an answer, the ambiguity disappears.’’ [29, p. 8]

Clearly, forensic scientists should aim at a reporting language that offers more than qualitative vagueness. The way out of the impasse of qualitative wording, however, does not come effortlessly. It brings us back, as noted in the above quote, to the fundamentals of the scientific approach to uncertainty measurement, which is probability.

A further way to understanding this argument is to ask: Why do we need to hire an expert? The answer is that one resorts to experts whenever one seeks an assessment for an issue that lies beyond one’s area of expertise. Experts’ assessments can take many forms, of course, with the lowest level of assessment often being regarded as one coarsely referring to possibilities. However, we should ask how useful a mere statement of possibility is. Suppose that all an expert would be able to say is that it is possible to observe this evidence if proposition A were true, but that is also possible to observe the same evidence in the event that another proposition, B, were true. To be clear, there are situations in which this is all that can meaningfully be stated – i.e. a case in which the evidence would have no probative value – but this should not be taken to mean that reporting language in terms of mere possibilities should set the bar. The truth is that it is by quantification that we make our way through the world: at the end of the day, humans in their professional activities as well as in other matters regarding their daily lives must make decisions whose consequences depend on uncertain states of nature, that is probability. This is not to say that a probability needs to be framed in full quantitative terms (i.e. numerically). An order of magnitude may be sufficient to be useful [e.g., 28].^42^ Contrast this with an expression in terms of possibility which means nothing more than a probability different from zero or one.

To illustrate this point, suppose being told that you won the lottery. This may be little helpful for you as your immediate question will be ‘How much?’. Suppose further that the answer you receive is something like ‘a lot’, ‘sufficiently much’, etc. At this point, you may end up being annoyed and ask ‘How do you know what *a lot* means *for me*? – Give me the number!’.^43^ This is also the reason why scales for the value of evidence, e.g. as proposed in Ref. [68,80], start from numbers and relate them to words, not the reverse [7,42,67]. DECDs, in turn, start and end with words. This is not to claim that well-recognised value of evidence scales [68,80] are a panacea for reporting problems in forensic science: indeed, if there is a number, or at least an order of magnitude, it becomes questionable whether there is any (added) value in couching this expression in verbal terms for which no uniform understanding across individuals can be assumed.

To summarise, let us clarify what exactly we argue for. We are *not arguing* that in a rationality-driven world, everyone should necessarily use probability in a fully numerical or computational way. Often, targeting an advanced level of detail or consideration of a densely related collection of variables will pose operational difficulties. Practical decisions are typically responsive to many variables, thus making computation come at the price of efficiency, or even worse: analysis paralysis. We also make no prescriptive claim of how exactly evaluative reporting should be formulated in practice (but see examples given in Ref. [80]). Our concern in this paper is the forensic scientists’ mindset which we consider a necessary preliminary to subsequent practical proceedings. Indeed, forensic science has been referred to as a state of mind because ‘‘(…) whether a particular individual is behaving, at a given juncture, as a scientist can be determined by the mental processes underlying his/her actions and words’’ [32, p. 121].

What we *are* saying is that probabilistic expressions can take the form of both numbers and words. The most important evidential test in law, that of relevance, depends on the tendency of an evidence item to make a proposition more or less probable. Probabilistic judgments by participants of the legal process, whether on relevance, degree of persuasion or aspects of forensic expertise, and whether numerical or verbal, have a quantitative connotation. The role of numbers is to substantiate comparisons. Hence, DECDs decidedly fall short of the point. What matters, though, is the way they miss the point. As outlined above, the proposed verbal descriptors are not inherently useful or useless. The insurmountable problem is, we think, that these six descriptors do not really describe anything insofar as they lack grammar. They are void of rules or standards of usage despite their ad-hoc description given in Table 1 [in Ref. [45]]: anyone’s take on ‘‘persuasive’’ or ‘‘conceivable’’ will be as (in-)valid as anyone else’s.^44^ Criteria of conformity and completeness are structural properties (see also [6, p. 29–37]), they cannot be subsumed within surface features of explanation as suggested by the DECDs framework.

## 5 Discussion and conclusions

A new expression, ‘digital transformation’, has become fashionable and is currently circulating in forensic science. It is associated with forensic science encountering novel forms of evidence, especially digital evidence. It is argued that such evidence implies distinct challenges. For example, Horsman mentions ‘‘the nature of digital evidence and investigations of this type’’ [p. 3], in particular ‘‘intricacies of digital data’’ [p. 1], as reasons why ‘‘a scientific mechanism for quantifying digital evidence may not actually be feasible’’ [p. 3]. He also argues that ‘‘attempts to quantify any uncertainty should be abandoned’’ [p. 1]. As shown throughout the present paper, this viewpoint is wide of the mark, and misconceives the notion of probability on several fundamental aspects. The discussion in this section seeks to summarise two main points and place them in a wider perspective. This will lead to conclusions that depict an alternative view on what the pending challenges in (digital) forensic science are.

The first argument advanced here is that the scientific measure of uncertainty, probability, is a liberal and flexible concept that can be conceptualised in largely untechnical terms.^45^ Challenges arise, even enthusiastic supporters of probabilism do not deny, when applying probability. That is, when eliciting a person’s probability regarding an event about which the person is uncertain. But the crucial point here is that an applicational challenge is not detrimental to the logic of the method. As lawyers know all too well, and they have stressed repeatedly, the fact that the world is a complicated place is neither a problem nor the fault of the method [e.g., 37]. The limits lie with their user and, indeed, ‘‘any theory that could not in principle represent the complexity surrounding us would have limited value’’ [38, p. 288]. Also, it should go without saying that conceptual frameworks do not work in a void. On the contrary, they need to be placed into context through supporting argument [54]. What these observations show us is that the primary problem does not necessarily rely with the forensic substance matter, that is (digital) evidence, however intricate it may be. Instead, the prospect of progress in digital forensic science critically hinges upon the (commitment to the) proper understanding of primary concepts related to the nature, purpose and use of formal methods of reasoning applied to the assessment of probative value. From a historical perspective, this is not a novel conclusion. Already decades ago, it has been noted that ‘‘no science is without some mathematical background, however meager’’ [56, p. 435], yet in forensic science, ‘‘[m]ost, if not all, of the amateurish efforts of all of us to justify our own evidence interpretations have been deficient in mathematical exactness and philosophical understanding’’ [56, p. 436]. What this means for digital forensic science is potentially far-reaching: as a currently developing new branch of forensic science, it has a unique opportunity *not* to commit the failures and shortcomings in evidence interpretation that (continue to) affect traditional forensic disciplines. However, this would require the community of researchers and practitioners to draw suitable conclusions from the principles of forensic interpretation that have been developed since the middle of the last century. The DECD conclusion scale that replicates an inferentially problematic design seen previously in forensic science (exposed here in Section 4), along with critiques levelled against probability also seen elsewhere in literature, are not conducive of such a perspective.

The second strain of argument, related to the above, calls for clarity on the purpose and the properties of primary concepts, in particular forensic interpretation and evaluative reporting as opposed to, for example, investigation (see also [76]). From a methodological point of view, an analytical method is needed because, firstly, probative value is not an intrinsic property of an item of evidence that somehow exists independently of a human interpreter, and, secondly, the purpose of interpretation at the evaluative stage is to try to work out and assign meaning. The concept of Digital Evidence Certainty Descriptors (DECDs) falls short of this; as its name reveals, it is essentially descriptive. Though this is not to say that one cannot be descriptive using analytical concepts, which includes probability as a scientific measure of uncertainty; we simply want to stress that this is not what Horsman promotes. As mentioned above, he argues to the contrary, i.e. that ‘‘attempts to quantify any uncertainty should be abandoned’’ [p. 1]. This is an opinion to which one may be entitled, but it is worthwhile for the readership to understand what the wholesale rejection of methods for the quantification of uncertainty, in particular probability, means – from a scientific perspective. The explanation of this requires a brief detour into the history of science. According to Cohen, science saw a ‘‘probabilizing revolution” [22, p. 324] at the beginning of the twentieth century. It involved developments in physics, chemistry and life sciences, such as genetics. The probabilizing revolution, in this context, designates the common thread among these science disciplines (including the social sciences) that consisted of the introduction of ‘‘a set of theories and explanations that were based on probability’’ [22, p. 96]. This was about a hundred years ago. Science has evolved since then with probability now being an integral and natural part of it, a fact that most contemporary commentators would not even consider necessary to emphasise. Thus, by calling for an abandonment of attempts to quantify uncertainty, Horsman suggests that the mindset of (digital) forensic scientists is to be equipped with less than what the state of *science* was a century ago.

How can a forensic branch get this far? In our view, a recurrently invoked pattern of argument to avoid engagement with primary concepts is claiming exceptional status. It has already been observed elsewhere before in forensic science and we also see it nowadays occurring in the context of digital forensic evidence. The claim, however, that digital forensic science is inherently different from other forensic branches that abide by scientific principles does not meet the *normative* requirements, both methodological and procedural in nature, which forensic fields *ought* to fulfill. A set of descriptors that lack an underpinning conceptual framework which could regulate usage in practice is sidestepping the requirement for justification. A lack of accountability represents a loss, as it removes justifications and reasoning processes entirely from the public arena. If this is what (digital) forensic science is or aims at, then it is difficult to see how it can meaningfully serve the needs of fact-finders in the pursuit of justice.

## Declaration of competing interest

The authors declare that this paper was written in the absence of any commercial or financial relationships that could be construed as a potential conflict of interest.

## Footnotes

1 Evidence of the transformation of forensic science is the fact that, as of 2020, the former journal ‘Digital Investigation’ is continued as ‘Forensic Science International: Digital Investigation’ [19].

2 From now on, for shortness of notation, all direct quotes from Horsman’s paper [45] are given without specific reference, only with page numbers.

3 Broadly speaking, propositions are statements about possible states of the world (or, events therein). Propositions are either true or false and can be affirmed or denied (see also [80, p. 24]).

4 Throughout this paper, the terms (forensic) scientist, examiner and expert are coarsely treated as synonyms.

5 As we will explain in later sections of this paper, the requirements for evaluative reporting should to be distinguished from those of investigative reporting.

6 We note, however, that exceptionalism also manifests itself in forensic science at large. Lander [58], for example, critically exposes exceptionalism in the ANZFSS council statement on the President’s Council Of Advisors On Science And Technology Report [70]. For further examples in scientific literature, see Section 2.1, in particular footnote 10.

9 See e.g. Aitken et al. [1] (with reference to earlier works by I.J. Good in the 1950s) on an argument for the inevitability of the likelihood ratio for the assessment of uncertainty inherent in evidence evaluation, and [43] for a rebuttal from a range of experts in forensic interpretation.

10 Unsupported claims of disciplinal distinctiveness are recurrently raised in many areas of forensic science. For an example of the claim that probability is not applicable in the area of forensic pattern analyses, see [51, 52] and related comments in [10, 12].

11 The expression ‘‘create a level of uncertainty’’ [p. 3] begs the question (and causes confusion) about the ‘‘uncertainty’’ about what and by whom. It would seem, from further quotes on the same page, that the author refers to the uncertainty on the part of the expert about the truth of a proposition or explanation (technically, a posterior probability). This reveals a difficulty in understanding forensic inference and the expert’s role. We will come back to this in the last paragraph of Section 2.6, insisting on the importance of clarity of, and agreement on, language.

12 In what follows, only ‘your uncertainty’ will be used for shortness of notation and as a way to make the exposition more appealing to the reader.

13 For reasons explained in [9, 13], relative frequency is not directly amenable to measuring uncertainty, though may inform it.

14 The device is a general one and nothing prevents it from being applicable in other contexts where events *can* be considered as instances of a series of like events, and relative frequency data may be available. But, even in those cases, relative frequency data does not define probability, it merely informs it [65, 75]. By separating the definition from the evaluation of probability, belief-type probability avoids the drawbacks of classic accounts that attempt to devise a single criterion based on, for example, frequency or symmetry [40].

15 Note that there are other ways to arrive at probability as a measure of uncertainty. One such other way, mentioned briefly later in Section 2.5, is based on scoring rules and considering the assignment of a probability as a personal decision. For a summary of different methods of establishing the rules of probability, see Lindley [65, p. 74–75].

16 See Section 4.1 for further discussion on the difference between description and analysis.

17 Note that only the draw of a single ball is to be contemplated, and the outcome of this draw is determined by the proportion of black balls. In particular, there is no repetition involved here (i.e., repeated drawing of balls) and no idea of a relative frequency of coloured balls. Literally, ‘‘[a]fter its withdrawal, the urn and its contents can go up in smoke for all that it matters.’’ [65, p. 36].

18 One such other device is the spinning of a wheel with coloured segments of varying size. Here, the probability of the wheel stopping on a coloured segment depends on the size of the segment, that can be adjusted, in analogy to the adjustable proportion of coloured balls in a bag, so as to reflect one’s uncertainty. See [78] for further discussion, and [50] for an application to probabilistic reasoning for lawyers.

19 As an aside, this defies the false claim that a probability can only be assigned if there are hard data.

20 Note that even though this quote refers to decision-making, it is suitable for the current discussion of uncertainty measurement using probability because probability assignment is an integral part of coherent decision analysis.

21 Probability is a liberal concept in the sense that it involves rather few constraints, such as coherence.

22 See [17] on the notion of justified subjectivism, and [13] for a detailed discussion of common misconceptions about probability.

23 See Section 2.5 for an understanding of probability assignment as a decision that can help reinforce the idea that scientists take responsibility for their assessments.

24 See Section 4 on further details about this framework.

25 Note that the ENFSI Guideline specifically mentions sensitivity analysis as a device for use by forensic examiners (’‘forensic practitioners should consider exploring the sensitivity of the likelihood ratio to different probabilities’’ [80, p. 9]).

26 See Section 4 for further details on this framework.

27 See Section 3 for more on the notion of evaluative reporting.

28 See, however, [13, 14, 16] for a discussion of the relevance of these topics for expert probability reporting in forensic science.

29 Another method, based on a standard, has been introduced previously in Section 2.2.

30 See Section 3 on the difference between the probability of the findings given the proposition, and the reverse, i.e. the probability of a proposition given evidence.

31 In specialised literature, notation such as ℓ(H|E) is sometimes used to denote the likelihood of hypothesis *H* given evidence *E*, which is the same as the Pr(E|H), the probability of *E* conditional on *H* (see, e.g., [2]).

32 It must be stressed that there are potentially severe limitations to investigative opinions, not least of which is tunnel-vision.

33 It remains legitimate to ask, though, whether even in investigation examiners should restrict their reporting to the findings only [e.g., 20], so as to leave investigative authorities form their own conclusions based on all elements on a case including other, non-technical/scientific items of evidence.

34 Though it is possible, of course, to develop probabilistic accounts for investigative purposes [e.g., 74].

35 Among these key messages is the precept, advised against by Horsman, of attaching to the probability of the evidence given the proposition: ‘‘(…) the single most important advance has nothing to do with technology (…). It tells us the most important lesson for the logic of evaluative forensic science: *consider the probability of the evidence, given the proposition*.’’ [33, p. 159].

36 Note that the (probative) value of the evidence is a function of the probability of the evidence given propositions [e.g., 1, 39].

37 The evidence is uninformative in such a case because there is no statement about its probative value.

38 *Golds* [2016] UKSC 61, para 37. See also *Brutus v Cozens* [1973] AC 854.

39 Not also that there is technical argument to show that direct assertions about propositions are not amenable for logical combination, in particular in the context of the joint assessment of multiple items of evidence (see [72] for a discussion).

40 While we agree generally with this comment that explanations are often ‘‘obvious’’, we acknowledge that there are situations in which an explanation is quite technical and would not be obvious to the lay person.

41 With the exception noted in footnote 40.

42 See Section 2.4 for further references on qualitative probabilistic reasoning.

43 The interested reader can find more discussion on numbers in [5, 59].

44 Note that this also affects other forensic value of evidence scales [67] proposed, for example, in [68, 80].

45 The particular conceptual device introduced in Section 2.2 requires little more than thinking in terms of proportions.

## Footnotes

7 As an aside, note that this is improper wording. What is being quantified is not the ‘evidence’, but the *weight* of evidence.

8 Note also that the primary role of formal methods of reasoning is not to merely *describe* – as Horsman suggests – the value of evidence, but to help elicit, assign and assert it. Stated otherwise, interpretation is analytical, not descriptive. See further discussion of this in Section 4.
